# Association of Opioid Consumption Profiles After Hospitalization With Risk of Adverse Health Care Events

**DOI:** 10.1001/jamanetworkopen.2021.8782

**Published:** 2021-05-18

**Authors:** Siyana Kurteva, Michal Abrahamowicz, Tara Gomes, Robyn Tamblyn

**Affiliations:** 1 Department of Epidemiology and Biostatistics, McGill University, Montreal, Quebec, Canada; 2 Clinical and Health Informatics Research Group, Department of Medicine, McGill University, Montreal, Quebec, Canada; 3 Institute of Health Policy Management and Evaluation, Toronto, Ontario, Canada; 4 Li Ka Shing Knowledge Institute, St Michael's Hospital, Toronto, Ontario, Canada; 5 ICES, Toronto, Ontario, Canada; 6 McGill University Health Centre, Montreal, Quebec, Canada

## Abstract

**Importance:**

Although better pain management has guided policies for opioid use over the past few decades, evidence is limited regarding how patterns of use are associated with the risk of potentially avoidable opioid-related adverse events.

**Objective:**

To estimate the risk of harms associated with opioid dose and duration of use, and to ascertain whether the risk is modified by treatment indication and age.

**Design, Setting, and Participants:**

This ad hoc cohort study followed up patients who were enrolled in a cluster randomized trial of medication reconciliation between October 1, 2014, and November 30, 2016, 12 months after they were discharged from the McGill University Health Centre in Montreal, Quebec, Canada. To be eligible for this study, patients needed to have filled at least 1 opioid prescription 3 months after discharge. Patients with a history of using methadone or buprenorphine were excluded. Data analyses were performed between February 1, 2019, and February 28, 2020.

**Exposures:**

Time-varying measures of opioid use included current use, daily morphine milligram equivalent (MME) dose, cumulative and continuous use duration, and type of ingredients in prescription opioids used. Hospitalization records, dispensed prescriptions records, and postdischarge interviews were used to evaluate adherence to the opioid prescriptions after discharge.

**Main Outcomes and Measures:**

Opioid-related emergency department visits, hospital readmissions, or all-cause death. Outcomes were ascertained using provincial medical services claims and hospitalization databases.

**Results:**

Of 3486 participants in the cluster randomized trial (mean [SD] age of 69.6 [14.9] years; 2010 men [57.7%]), 1511 patients were included in this ad hoc cohort study. Among those with at least 1 opioid dispensation, 241 patients (15.9%) experienced an opioid-related emergency department visit, hospital readmission, or death. Results from marginal structural Cox proportional hazards regression models showed more than a 2-fold increase in the risk of opioid-related adverse events associated with a cumulative use duration of more than 90 days (adjusted hazard ratio, 2.56; 95% CI, 1.25-5.27) compared with 1 to 30 days. A 3-fold risk increase was found with a mean daily dose higher than 90 MME (adjusted hazard ratio, 3.51; 95% CI, 1.58-7.82) compared with 90 MME or lower.

**Conclusions and Relevance:**

This study found an association between risk of adverse health care events and higher opioid doses and longer treatment duration. This finding can inform policies for limiting opioid duration and dose to attenuate the risk of avoidable morbidity.

## Key Points

**Question:**

Is the risk of opioid-related emergency department visit, hospital admission, or death associated with the prescribed opioid dose and duration of use?

**Findings:**

In this cohort study of 1511 patients who were discharged from an academic hospital, both cumulative opioid use duration of more than 90 days and daily opioid dose higher than 90 morphine milligram equivalents were associated with increased risk of opioid-related adverse events.

**Meaning:**

Results of this study suggest that practitioners need to focus on adjusting pain management strategies for patients who are transitioning from acute postoperative to chronic pain and that policies should limit opioid use duration and doses to minimize opioid-related morbidity.

## Teaser

This cohort study assesses the association of duration, dose, current use status, and type of ingredients in prescription opioids consumed by patients before and after hospital discharge with risk of opioid-related adverse health care events.

## Introduction

Over the past 20 years, opioid prescribing and average prescription volumes continued to increase in the United States and Canada.^1,2^ Opioids remain the main treatment for cancer pain, as recommended by the World Health Organization.^3^ However, substantial increases in prescriptions for chronic noncancer pain have also been documented. In the 2010s in North America, opioid use increased by nearly 100%,^4^ with acute pain being the most common indication.^5,6^ These patterns in prescription opioids have been accompanied by higher rates of opioid-related morbidity and mortality.^4,7^ Nonfatal opioid-related outcomes have been reported in older adults, even when the drugs were used as directed.^8,9^ Furthermore, the long-term benefits are uncertain given that even short-term use may lead to greater predisposition to adverse events.^10^ Longer trials have shown less pain relief with opioids, possibly because of pain tolerance or opioid-induced hyperalgesia.^11^ This response may play a role in an escalation in the dose and potency of opioids, which subsequently may be associated with higher risk for adverse reactions. Yet, no opioid trial has followed up patients for longer than 6 months,^12^ and most observational studies have examined only the association between the initial dose of opioid prescriptions and the duration of subsequent use.^13,14,15^

For many patients, their first opioid exposure follows a hospitalization, making this group a high-priority population for investigation. Inadequate postdischarge communication of hospital-initiated changes in medication among community-based practitioners is a well-established problem.^16^ Consequently, community physicians may continue prescribing opioids started in the hospital for acute pain relief because they have no information on the treatment indication or the expected therapy duration. Thus, the prescribing practices during hospitalization may have implications for the increase in opioid consumption and its related adverse outcomes.

In this cohort study, we aimed to estimate the risk of harms associated with opioid dose and duration of use. We also aimed to ascertain whether the risk is modified by treatment indication and age.

## Methods

Ethics approval for this study was obtained from the McGill University Health Centre (MUHC) Research Ethics Board. Some patients provided written informed consent, whereas others provided verbal consent on the telephone. Privacy Commissioner permission to link clinical and administrative data was obtained from the Commission d’Accès à l’Information du Quebec. We followed the Strengthening the Reporting of Observational Studies in Epidemiology (STROBE) reporting guideline.^17^

### Design, Setting, Participants, and Data Sources

We followed up a prospective cohort of patients 12 months after their discharge from a medical or surgical unit. These patients were enrolled in a cluster randomized trial of medication reconciliation at the MUHC between October 1, 2014, and November 30, 2016.^18^ The MUHC is a quaternary care teaching hospital with more than 1000 beds in Montreal, Canada, that operates within the universal health care system of the province of Quebec (Régie de l’Assurance Maladie du Québec [RAMQ]). The RAMQ plan covers all hospitalizations, essential medical care, and drug insurance for registrants who are aged 65 years or older, are recipients of income security, and are not insured through their employer (approximately 50% of the Quebec population).

To be eligible for the original cluster randomized trial, patients had to be aged 18 years or older at admission, have been admitted from the community or transferred from another hospital, and have had at least 1 year of previous continuous provincial health care coverage. To be included in the present ad hoc study, patients needed to have filled at least 1 opioid prescription in the 90 days after discharge. We excluded patients with a history of using methadone hydrochloride or buprenorphine hydrochloride, which are prescribed to treat opioid addiction.^19^ eFigure 2 in the Supplement shows the patient selection for the study.

We linked multiple data sources. Demographic, clinical, health care use, and prescription claims data were retrieved from admission notes and from RAMQ medical services and prescription claims in the year before and after the index hospitalization. Data on admission and discharge dates, units, and diagnoses as well as major procedures were retrieved from the hospitalization database. Medications at admission, in hospital, and prescribed at discharge were abstracted from the MUHC Data Warehouse. Information on hospital discharge experiences was obtained through telephone interviews 30 days after discharge.

### Postdischarge Opioid Use 

Opioid use 1 year after discharge was measured using RAMQ pharmacy claims. For each prescription filled, the drug identification number, strength, dispensing date and quantity, prescription duration, and prescribing physician are documented in these claims. Drug identification numbers that were mapped to Anatomical Therapeutic Chemical codes N02A and R05DA were used to identify opioids (eMethods 1 in the Supplement). On each day, an individual was classified as having an active prescription or not. A 5-day grace period was added to the end of each dispensation given that opioids were often prescribed on a use-as-needed basis, and patients were likely to take some unused pills for a few days after the prescription ended. eMethods 2 and eTable 6 in the Supplement describe the opioid daily dose calculations.^20^ We also constructed time-varying binary indicators of recent opioid discontinuation, dose increases, and opioid add-on therapy.

This study was unique in that administratively derived measures of opioid exposure were supplemented with information on the patient’s medication-use behavior. This information was extracted from interviews 30 days after discharge to construct a series of time-invariant indicators that identified patients who filled a prescription and (1) continued using it, (2) started using it but stopped, or (3) never used it.

### Outcomes, Potential Modifiers of Harms, and Covariates

Outcome was defined as the time from the first opioid dispensation to the earliest of the first opioid-related emergency department (ED) visit, hospital readmission, or death from any cause in the year after discharge. Outcomes were ascertained using RAMQ medical services claims and hospitalization databases, which identified all ED visits and readmissions to any hospital. An adverse event was considered opioid-related if the diagnosis indicated opioid abuse, opioid dependence, and/or opioid poisoning and/or any of the more common adverse effects of opioids,^21^ including constipation, nausea, vomiting, dizziness,^22,23,24,25,26^ or fractures.^27,28,29,30^ We combined all possible opioid-related adverse effects because only 3 patients (0.2%) were diagnosed with opioid abuse, dependence, and/or poisoning and because medication-related adverse events are underascertained in the ED.^31^ eTables 1 to 5 in the Supplement list the *International Classification of Diseases, Ninth Revision, Clinical Modification*, and *International Statistical Classification of Diseases, Tenth Revision, Clinical Modification* codes we used.

We assessed potential modifiers of harms associated with opioid use, stratified by age (<64 years vs ≥64 years) and treatment indication, categorized as (1) cancer-related pain, (2) postsurgical pain management, or (3) other chronic pain problems. Medical records were used to identify treatment indication.

Potential risk factors for long-term opioid use included patient demographic characteristics (age, sex, and drug insurance status), coexisting illnesses (history of mental health conditions, pain syndromes, substance and/or alcohol use disorder, tobacco use, cancer diagnoses, and other comorbidities), and drug and health care use 1 year before hospitalization (opioid misuse; nonopioid pain medications; and number of ED visits, hospitalizations, physicians seen, and dispensing pharmacies). We also assessed the reason for hospital admission, in-hospital administration of opioids, the reason for opioid prescribing at discharge, whether a multimodal pain management regimen was prescribed, and discharge destination.^11,32^ To measure health care fragmentation associated with low quality of care and increased adverse outcomes or opioid-seeking behavior associated with greater dependence,^33,34^ we created time-varying covariates, updated daily during follow-up, for the cumulative numbers of distinct prescribers and dispensing pharmacies after discharge.

### Statistical Analysis

Descriptive statistics were used to summarize patient, practitioner, and health care system characteristics. For all main analyses, we relied on time-to-event methods. Specifically, we used multivariable marginal structural Cox proportional hazards regression models (MSM Cox)^35,36^ to identify the association between time-varying opioid use and the risk of the outcome. Cohort entry was the date of the first opioid dispensation within 3 months after hospital discharge. Patients were followed up until their first opioid-related ED visit, hospital readmission, death, or end of follow-up. We temporarily censored patients during hospitalizations that were unrelated to opioid use. Because of the uncertainty regarding the association of opioid consumption patterns with adverse events, we constructed alternative time-varying metrics of opioid use, which were updated daily during the 1-year follow-up period: (1) current use (no or yes), (2) cumulative use duration (1-30, >30-60, >60-90, and >90 days), (3) continuous use duration (0, 1-30, >30-60, and >60 days), (4) daily dose in morphine milligram equivalent (MME; ≤90 or >90), and (5) type of ingredient in prescription opioids used (codeine sulfate, morphine sulfate, oxycodone hydrochloride, hydromorphone hydrochloride, fentanyl citrate, or multiple opioid products) (eTable 7, eFigure 1, and eMethods 3 in the Supplement).^37^

All MSM Cox models included the same potential confounders, including time-invariant baseline variables and time-varying covariates, that were selected according to statistical and clinical significance. To account for time-varying potential confounders that could also be affected by previous opioid exposure, we used psychotropic medication use, targeted comorbidities, and cumulative numbers of prescribers and dispensing pharmacies to estimate stabilized time-varying inverse probability treatment weights^38,39^ for opioid exposure. To estimate the inverse probability treatment weights, separately for each 10-day time interval during the 1-year follow-up, we used a series of multivariable logistic models,^39^ and to avoid unstable estimates, we truncated at the 95th percentile of their distribution.^40^ We used the robust sandwich covariance estimator to calculate SEs, while accounting for the inverse probability treatment weights.^41^ To ascertain whether the risk of opioid-related harms was modified by treatment indication or age, we used Wald tests of the respective 2-way interactions at 2-sided α = .05.

All MSM Cox models were implemented with SAS, version 9.4 (SAS Institute Inc). Data analyses were performed between February 1, 2019, and February 28, 2020.

### Sensitivity Analyses

First, to account for potential long-term opioid use before study entry, we recreated the cohort in 2 sensitivity analyses, excluding patients with 1 or more opioid dispensations from the first analysis and those with 3 or more opioid dispensations from the second analysis, during the baseline period. Second, in separate sensitivity analyses, we restricted the outcome to either opioid-related ED visit, hospital readmission, or death. We also conducted sensitivity bias analyses in which we tested an interaction term between the main exposure and the adherence measure constructed from patient interviews to identify the extent to which the potential nonadherence to opioid prescriptions could have affected the estimated association. Third, to account for the differences in severity of opioid-related adverse effects, in 2 additional sensitivity analyses, we categorized the outcomes into fracture or dizziness and nausea or constipation.

Additional sensitivity analyses were performed to ascertain the extent to which selected, statistically significant results could reflect potential bias from unmeasured confounders.^42^ The analyses involved (1) simulating a potential confounder with prespecified associations (odds ratios) with both relevant opioid exposure and occurrence of the outcome and (2) rerunning the multivariable analyses with additional adjustment for the simulated confounder.^43^

## Results

Among the 3486 participants in the original cluster randomized trial, including 2010 men (57.7%) and 1476 women (42.3%) with a mean (SD) age of 69.6 (14.9) years (Table 1), 1511 were followed up in the current study. Most patients underwent surgery (1119 [74.1%]), and 392 (25.9%) received care in the medical unit. At hospital discharge, 202 of 392 patients (51.5%) from the medical unit and 987 of 1119 patients (88.2%) from the surgical unit received an opioid prescription. A list of the prescriptions dispensed by type of opioid ingredient and potency is provided in Table 2.^20,44^ Among the 348 patients who did not receive an opioid prescription at discharge, 178 (51.2%) filled an opioid prescription in the 7 days after discharge (Table 3). Fewer surgical than medical patients used opioids before admission (344 of 1119 [30.7%] vs 283 of 392 [72.2%]). Overall, 168 of all patients from the medical unit (42.9%) and 538 of all patients from the surgical unit (48.1%) had documented cancer diagnoses in the year before hospitalization and/or at hospital discharge. In the year after discharge, 241 patients (15.9%) had an opioid-related ED visit or hospitalization or died. The most frequent potentially opioid-related adverse events were fractures (219 [51.8%]), nausea and vomiting (66 [15.6%]), and dizziness (78 [18.4%]). Additional results from descriptive analyses and main models are shown in eTables 8 to 17 in the Supplement.

**Table 1.** Baseline Characteristics of Patients Who Filled an Opioid Prescription Within 90 Days of Hospital Discharge, Stratified by Discharge Unit

Characteristic	Trial cohort, No. (%)	Follow-up cohort, No. (%) (n = 1511)		
		Discharged from medical unit		Discharged from surgical unit
All patients	3486	392 (25.9)		1119 (74.1)
Age, mean (SD), y	69.6 (14.9)	67.7 (16.8)		66.9 (11.9)
Female	1476 (42.3)	192 (49.0)		436 (39.0)
Male	2010 (57.7)	200 (51.0)		683 (61.0)
Length of hospital stay, ≥6 d	2930 (84.0)	351 (89.5)		875 (78.2)
Health care use 1 y before admission, mean (SD)				
ED visits	8.4 (8.5)	15.3 (20.3)		4.4 (8.1)
Hospitalizations	0.8 (1.9)	0.9 (1.9)		0.7 (1.8)
Physician visits	10.9 (14.5)	14.0 (16.3)		9.9 (8.4)
No. of prescribing physicians	4.2 (3.4)	6.3 (4.4)		3.6 (2.4)
No. of physicians prescribing opioids	0.6 (1.2)	1.9 (2.2)		0.5 (0.9)
No. of dispensing pharmacies	1.4 (0.9)	1.6 (0.9)		1.4 (0.8)
No. of pharmacies dispensing opioids	0.4 (0.6)	0.9 (0.7)		0.4 (0.6)
Active prescriptions at admission	9.8 (10.1)	14.3 (14.1)		6.6 (6.5)
Radiotherapy	215 (6.2)	77 (19.6)		133 (11.9)
Chemotherapy	262 (7.5)	83 (21.2)		176 (15.7)
Medication use 1 y before admission				
Active opioid prescription at admission	504 (14.5)	186 (47.4)	105 (9.4)	
History of opioid use	1206 (34.6)	283 (72.2)	344 (30.7)	
≥3 opioid dispensations	104 (2.9)	61 (15.6)	18 (1.6)	
History of long-acting opioid use	146 (4.2)	89 (22.7)	35 (3.1)	
History of methadone or buprenorphine use	13 (0.4)	10 (2.6)		1 (0.1)
History of benzodiazepine use	1088 (31.2)	175 (44.6)		336 (30.0)
History of antidepressant use	706 (20.3)	133 (33.9)		208 (18.6)
History of nonopioid pain medication use	1068 (30.6)	243 (61.9)		406 (36.3)
In-hospital medication use				
Antidepressants	628 (18.0)	112 (28.6)		153 (13.7)
Opioids	2509 (72.0)	307 (78.3)		1113 (99.5)
Benzodiazepines	2278 (65.4)	196 (50.0)		997 (89.1)
Analgesics	3161 (90.7)	168 (43.1)		942 (84.2)
Pain regimen at hospital discharge				
Opioids	1530 (43.9)	202 (51.5)	987 (88.2)	
Nonopioid analgesics	2209 (63.4)	227 (57.9)	990 (88.5)	
Targeted comorbidities that may increase the risk of hospitalizations or ED visits				
History of mental illness	511 (14.7)	74 (18.9)		132 (11.8)
Dementia	213 (6.1)	25 (6.4)		13 (1.2)
Substance and/or alcohol use disorder	115 (3.3)	27 (6.9)		19 (1.7)
Pain syndromes	1352 (38.8)	221 (56.4)		408 (36.5)
Cancer diagnosis	1253 (35.9)	168 (42.9)		538 (48.1)
Other comorbidities that may increase the risk of hospitalizations or ED visits				
Cardiovascular diseases	1398 (40.1)	154 (39.3)		657 (58.7)
Cerebrovascular diseases	334 (9.6)	49 (12.5)		69 (6.2)
Pneumonia	338 (9.7)	46 (11.7)		63 (5.6)
COPD	751 (21.5)	102 (26.0)		236 (21.1)
Kidney disease	364 (10.4)	53 (13.5)		41 (3.7)
Diabetes	791 (22.7)	92 (23.5)		223 (19.9)

Abbreviations: COPD, chronic obstructive pulmonary disease; ED, emergency department.

**Table 2.** Characteristics of Prescription Opioids Dispensed, Stratified by Ingredient Type and Potency^20,44^

Ingredient (molecule)^**a**^	MME conversion factor	Patients who filled at least 1 type of opioid ingredient, No. (%)^**b**^	Days’ supply of initial dispensation, mean (SD)	Dose of initial dispensation, mean (SD), MME	Patients who filled ≥2 opioid prescriptions, No. (%)	Patients who filled ≥1 type of opioid ingredients, No. (%)
Codeine sulfate	0.15	215 (14.2)	13.2 (10.1)	19.9 (12.4)	201 (93.5)	11 (5.1)
Morphine sulfate	1	244 (16.1)	10.4 (8.3)	27.4 (27.2)	226 (92.6)	159 (65.2)
Oxycodone hydrochloride	1.5	1044 (69.1)	8.6 (6.3)	35.3 (17.7)	610 (58.4)	441 (42.2)
Hydromorphone hydrochloride	4	689 (45.6)	9.8 (7.4)	31.8 (26.0)	594 (86.2)	180 (6.1)
Fentanyl citrate	7.2	109 (7.2)	22.5 (11.3)	137.2 (121.5)	108 (99.1)	16 (14.7)
Methadone hydrochloride	NA	44 (2.9)	NA	NA	37 (84.1)	33 (75.0)
Total No. of patients^**c**^	NA	1511	NA	NA	950 (62.8)	595 (39.4)

Abbreviations: MME, morphine milligram equivalent; NA, not applicable.

^a^ Only the tablet and patch forms of these medications were considered.

^b^ A given patient can be in more than 1 category as the patient fills multiple types of opioid ingredients.

^c^ This total is not equivalent to the sum of patients in each opioid ingredient category.

**Table 3.** Characteristics of First Opioid Prescription Filled in the 90-Day Postdischarge Period

Characteristic	Overall	Opioid prescription at discharge?	
		Yes	No
All patients	1511	1163 (76.9)	348 (23.0)
Opioid prescription filled			
Within first 7 d	1228 (81.3)	1050 (90.3)	178 (51.2)
Within first 30 d	1360 (90.0)	1118 (96.1)	242 (69.5)
MME dose dispensed			
Mean (SD)	34.9 (28.6)	34.9 (23.6)	34.8 (40.9)
Median (IQR)	29.1 (20.0-41.7)	30.0 (21.0-41.7)	25.0 (16.0-40.9)
MME dose dispensed			
≤90	1467 (97.1)	1137 (97.8)	330 (94.8)
>90	44 (2.9)	26 (2.2)	18 (5.2)
Type of opioid ingredients dispensed			
Codeine	47 (3.1)	21 (1.8)	26 (7.5)
Morphine	68 (4.5)	33 (2.8)	35 (10.1)
Oxycodone	952 (63.0)	814 (69.9)	138 (39.7)
Hydromorphone	419 (27.7)	286 (24.6)	133 (38.2)
Fentanyl	22 (1.5)	7 (0.6)	15 (4.3)
Combination opioid products dispensed	308 (20.4)	209 (17.9)	99 (28.5)
Combination nonopioid products dispensed	1300 (86.0)	999 (85.9)	301 (86.5)

Abbreviations: IQR, interquartile range; MME, morphine milligram equivalent.

Current opioid use, which identified patients as having an active prescription on a given day during the follow-up period, was associated with a 71% increased risk of opioid-related adverse events (adjusted hazard ratio [AHR], 1.71; 95% CI, 1.04-2.82) (Table 4). Compared with shorter cumulative exposure of 1 to 30 days, longer past use of more than 60 to 90 days (AHR, 2.45; 95% CI, 1.18-5.09) and more than 90 days (AHR, 2.56; 95% CI, 1.25-5.27) were both associated with a 2-fold increase in risk of adverse events. Uninterrupted continuous use for up to 60 days was associated with a 3-fold increased risk of opioid-related adverse health care events (AHR, 3.73; 95% CI, 1.83-7.60) compared with patients who were not current opioid users. In contrast, for a few patients who exceeded 60 days of continuous use, no evidence of an increased risk of adverse events was found (AHR, 0.86; 95% CI, 0.37-1.96).

**Table 4.** Risk of Adverse Events for Opioid Exposure Metrics in Marginal Structural Cox Proportional Hazards Regression Models

Opioid exposure metric	No. of adverse events^**a**^	Person-years	Incidence rate (95% CI)^b^	Risk of ED visits, hospital readmissions, or death, adjusted HR (95% CI)^**c**^^,^^d^	Risk of ED visits or hospital readmissions, adjusted HR (95% CI)^e^	Risk of death, adjusted HR (95% CI)
Current use						
No	128	1102.7	116.1 (96.8-138.0)	1 [Reference]	1 [Reference]	1 [Reference]
Yes	113	233.4	484.2 (399.0-582.1)	1.71 (1.04-2.82)	2.00 (0.98-4.10)	1.56 (0.79-3.04)
Cumulative use duration, d						
1-30	123	973.3	126.4 (105.0-150.8)	1 [Reference]	1 [Reference]	1 [Reference]
>30-60	44	181.1	242.9 (176.6-326.2)	1.55 (0.95-2.52)	1.47 (0.69-3.14)	1.61 (0.86-3.03)
>60-90	24	56.6	423.7 (271.5-630.4)	2.45 (1.18-5.09)	1.05 (0.22-3.92)	3.45 (1.41-8.47)
>90	50	125.1	399.8 (296.7-527.0)	2.56 (1.25-5.27)	2.07 (0.70-6.07)	2.89 (1.11-7.59)
Continuous use duration, d						
0	128	1102.7	116.1 (96.8-138.0)	1 [Reference]	1 [Reference]	1 [Reference]
1-30	63	132.0	477.3 (366.7-610.6)	1.79 (1.00-3.22)	2.10 (0.72-5.86)	1.66 (0.84-3.29)
>30-60	26	32.4	801.5 (523.5-1174.3)	3.73 (1.83-7.60)	5.19 (1.56-17.2)	3.10 (1.28-7.54)
>60	24	68.9	348.1 (223.1-517.9)	0.86 (0.37-1.96)	0.91 (0.32-2.49)	0.81 (0.26-2.47)
Daily MME dose						
≤90	207	1302.2	158.9 (138.0-182.1)	1 [Reference]	1 [Reference]	1 [Reference]
>90	34	33.9	1003.1 (694.7-1401.7)	3.51 (1.58-7.82)	1.06 (0.30-2.78)	5.84 (2.12-16.09)
Type of opioid ingredient used						
Codeine	4	13.5	296.9 (80.9-760.2)	1 [Reference]	1 [Reference]	1 [Reference]
Morphine	19	18.1	1047.5 (630.7-1635.8)	4.04 (1.02-15.9)	1.81 (0.28-11.6)	9.36 (1.18-73.9)
Oxycodone	16	78.8	202.9 (115.9-329.5)	1.48 (0.35-6.25)	0.67 (0.10-4.27)	2.98 (0.33-27.0)
Hydromorphone	45	87.2	515.8 (376.3-690.2)	2.62 (0.64-10.7)	1.06 (0.18-6.41)	6.74 (0.83-54.6)
Fentanyl	11	15.3	718.7 (358.8-1286.0)	2.93 (0.57-15.0)	0.43 (0.03-6.01)	8.67 (0.87-86.1)
Multiple opioid products	18	20.4	883.3 (523.5-1396.0)	6.36 (1.42-28.4)	4.74 (0.69-32.4)	9.94 (1.01-98.3)

Abbreviations: ED, emergency department; HR, hazard ratio; MME, morphine milligram equivalent.

^a^ The event counts are for the composite outcome of ED visits, readmissions, and/or death.

^b^ Incidence rate is reported as 1000 per year.

^c^ Covariates considered in the calculation of the inverse probability treatment weights were as follows: (1) demographic characteristics (ie, indicator for a patient randomized to the RightRx intervention group, age at admission, sex, and copay status); (2) medical, prescription, and health care use 1 year before admission (ie, unique number of dispensing pharmacies and prescribers, hospitalizations and ED visits, receipt of radiotherapy and/or chemotherapy services, type of cancer, history of mental health diagnoses, history of substance and/or alcohol abuse or dependence, targeted comorbidities that may increase someone’s risk of opioid-related adverse events, history of chronic pain, previous opioid use, more than 3 opioid dispensations, and previous use of psychotropic medications); (3) in-hospital characteristics (ie, presence of an opioid-related reason for index admission, length of hospital stay, opioid administration during the index hospitalization, nonopioid pain medication administration, use of antidepressants and benzodiazepines, hospital unit discharged from [medical vs surgical], and type of surgery [cardiac vs thoracic]); (4) at-discharge characteristics (ie, receipt of an opioid prescription; prescribing reason such as having had surgery, having anxiety, or pain problems); (5) time-varying postdischarge characteristics (ie, use of benzodiazepines, use of antidepressants, use of methadone or buprenorphine, cumulative number of physicians, cumulative number of dispensing pharmacies, recent discontinuation of opioid use, recent increases in opioid dose, recent add-on opioid therapy, and updated targeted baseline medical comorbidities). The 95th percentile for the stabilized weight was 2.88 (mean [SD] = 0.81 [0.71]).

^d^ The Akaike information criteria for the models were 2562.3 with current use, 2557.6 with cumulative use duration, 2548.1 with continuous use duration, 2535.5 with MME daily dose, and 2548.1 with type of opioid ingredient used.

^e^ Additional censoring weights were included to account for competing risk by death. Same covariates as those included in the treatment weights were used for the censoring weights.

The risk of opioid-related adverse events or death was 3 times higher for a current daily dose higher than 90 MME (AHR, 3.51; 95% CI, 1.58-7.82) vs 90 MME or lower. Among different types of opioid ingredients, only morphine showed statistically significant risk increase (AHR, 4.04; 95% CI, 1.02-15.9) compared with codeine, albeit with wide CIs (Table 4). We found 2 statistically significant interactions between surgery and current opioid use (AHR, 3.35; *P* = .003) and between surgery and more than 90 days of opioid use (AHR, 7.80; *P* = .002). Among patients discharged from the surgical unit, both current use (AHR, 3.35; 95% CI, 1.82-6.85) and cumulative use duration of more than 90 days (AHR, 7.80; 95% CI, 3.20-13.1) were associated with statistically significant increased risk of opioid-related adverse events or death. In contrast, both associations were not statistically significant for patients discharged from the medical unit. The interaction between cumulative opioid use duration of more than 90 days and a cancer diagnosis was also significant (eTable 13 in the Supplement). Results of interaction analyses by age and treatment indication are in eTables 12 and 13 in the Supplement.

Excluding previous opioid users slightly changed the results (eTables 9 and 10 in the Supplement). The absence of an interaction between adherence and current use (*P* = .99) could be attributed to excellent adherence: in the first month after discharge, 90% of patients (n = 1360) reported using their dispensed opioids as prescribed, only 12% (n = 169) discontinued their initial dispensation, and 5% (n = 70) filled their prescription but never started using it. In bias sensitivity analyses, patients with a daily opioid dose higher than 90 MME remained at a significantly increased risk of opioid-related adverse health care events, even after adjusting for a moderately strong unobserved confounder, with an odds ratio of 2 for both exposure (higher dose) and outcome (eTable 16 in the Supplement). Analyses that restricted the outcomes to either fracture-related or other opioid-related ED visits or hospital readmissions showed consistent results (eTable 17 in the Supplement).

## Discussion

We assessed the association between long-term opioid use patterns (represented by time-varying measures of current daily use, daily MME dose, cumulative and continuous use duration, and type of opioid ingredient) and opioid-related adverse events or death. We found increased risks with daily dose as well as with cumulative and continuous use duration. There were also variations in the magnitude of risk when considering different treatment indications. The results highlight the importance of accounting for alternative opioid consumption patterns when quantifying the risk of adverse health care events or death. Although much of the literature considers 90 days as a threshold for safe opioid use,^14,45,46^ in this study, we provided risk estimates for multiple duration categories of up to and beyond 90 days to illustrate how the risk of adverse events may vary with short-term and long-term use patterns.

Observational research into extended opioid treatment duration and associated adverse events is scant, with most of the evidence coming from clinical trials with a duration of less than a year and previous observational studies examining only the initial opioid prescription duration.^14,47,48^ Regarding the risks of opioid use duration, results of the current study are similar to the findings of a 2017 Cochrane Reviews summary.^12^ In this study, the nonsignificant findings and wide CIs for continuous use beyond 90 days could be a reflection of low statistical power given that only a few patients had long, uninterrupted use during the 1-year follow-up. However, it has been previously documented that just as patients developed tolerance to the analgesic properties of opioids, they also developed the capacity to tolerate adverse outcomes.^49^ In this study, mean opioid doses started to plateau by the greater than 90-day threshold and did not escalate with increasing duration beyond 90 days of use (eTable 13 in the Supplement), which suggests that these patients potentially transitioned into long-term users.

We noted an increased risk of adverse events with daily doses higher than 90 MME. Previous studies have also demonstrated an association of opioid dose with adverse events, such as increased risks of fractures, road trauma, and opioid-related mortality.^2,50,51,52,53^ Saunders et al^50^ showed that a daily opioid dose higher than 50 MME was associated with a 2-fold increase in the risk of fractures. Similar to our study, work by Ishida et al^54^ found high doses to be associated with risks for all adverse outcomes. The present study showed that use by most patients in this cohort did not exceed the recommended maximum dose of 90 MME,^32^ and yet their risk of adverse events was still high vs the risk of patients who were not exposed to opioids.

Existing data on the rates of morbidity and mortality as a function of drug potency among commonly prescribed opioids are somewhat conflicting.^55,56,57,58^ However, because of the relatively small sample size and overlapping CIs in this study, our comparisons of the risks associated with different prescription opioids were inconclusive, even if the results suggested that morphine users may be at higher risk of a composite outcome and death.

Results of this study can inform pain management policies or strategies aimed at preventing or attenuating opioid-related morbidity. Practitioners need to adjust opioid use duration and opioid doses for patients who are transitioning from acute postoperative to chronic pain.

### Strengths and Limitations

This study has some strengths. It used multiple data sources, which enhanced the internal validity of the study by providing detailed covariate information to adjust for confounders and account for potential mediators. Most of what is known about extended opioid treatment and associated adverse events is based on different and arbitrary definitions.^59,60,61,62^ In this study, we compared various time-varying opioid use metrics to provide further insights into the mechanism behind the development of opioid-related events.^37,63^

This study has some limitations. In the data analyses, we used prescription duration as recorded by pharmacists. Because opioids are given on an as-needed basis, exposure mismeasurement is possible. However, we expect the resulting exposure misclassification to be nondifferential and thus to bias the estimates toward the null. As in all observational studies, this study had the potential for unmeasured confounding and confounding by indication. Our decision to include only patients with at least 1 opioid dispensation after discharge (excluding never users) and to select as a comparator those patients on short-term or low-dose opioids reduces concerns about potential bias from confounding by indication. Moreover, a consistent limitation across all claims-based studies, including this study, is the inability to account for opioid medications that were obtained through diversion or other illicit means. However, a study conducted in a similar cohort of universally covered patients in the province of Quebec found older adults to be less likely to experience an opioid prescription–associated overdose death and to seek outpatient prescriptions compared with younger people. Most patients in this study cohort were 64 years of age or older; thus, we expected illicit use to have little implication for the main findings.^64^ In this study, the choice of a broader outcome may be prone to confounding. However, we explored the amount of hidden bias from a simulated confounder necessary to alter the conclusion that patients with higher daily opioid doses have a higher risk of opioid-related adverse events, and the association was robust. Future research using data from multiple health care systems is required to replicate these findings in larger population cohorts and provide greater generalizability.

## Conclusions

In this cohort study, we found that using opioids for prolonged duration and at high doses was associated with increased risk of opioid-related adverse events or death. These results can inform policies or strategies for minimizing the harms and risks associated with opioid-related morbidity. Opioid use duration and opioid doses may need to be adjusted for patients who are transitioning from acute postoperative to chronic pain.

## Notes

Supplement. **eTable 1.** ICD-9-CM and ICD-10-CM Codes for Opioid Abuse **eTable 2.** ICD-9-CM Codes for Opioid Dependence **eTable 3.** ICD-9-CM Codes for Adverse Effects of Opioids **eTable 4.** ICD-9-CM Codes for Opioid Poisoning **eTable 5.** ICD-9-CM Codes for Other Most Commonly Occurring Adverse Events Associated With Opioid Use **eMethods 1.** ATC Codes Used to Identify Opioids: N02A (Opioids), R05DA (Opium Alkaloids and Derivatives) **eMethods 2.** Daily Dose Calculation **eTable 6.** Opioid Morphine Equivalent Conversion Factor **eTable 7.** Description of Available Data on Drug, Patient, Provider and System Level Characteristics **eFigure 1.** Operational Definitions of Opioid Use Duration **eMethods 3.** Operational Definitions of Opioid Use Durations **eTable 8.** Overall Characteristics of the Opioid Prescriptions Dispensed by Patients According to Opioid Type and Potency **eTable 9.** Sensitivity Analyses Excluding Patients With More Than Three Opioids Dispensations in the One Year Before Initial Hospital Admission (Final Cohort N=1468) **eTable 10.** Sensitivity Analyses Excluding Patients With an Opioids Dispensation in the One Year Before Initial Hospital Admission (Final Cohort N = 884) **eTable 11.** Breakdown of the Reasons for the Healthcare Encounters in the One-Year Post-Discharge Among Patients With At Least One Opioid Dispensation **eTable 12.** Analyses for Current Opioid Use and Cumulative Duration of Opioid Use Based on Age **eTable 13.** Analyses for Current Opioid Use and Cumulative Duration of Opioid Use Based on Treatment Indication **eTable 14.** Results from Statistically Significant Additional Interactions Terms Between Current and Cumulative Duration of Opioid Use and Concurrent Use of Buprenorphine/Methadone and Benzodiazepines **eTable 15.** Characteristics of Patients in the Weighted Study Population According to the Receipt of an Opioid Dispensation at 10 Days Since Beginning of Follow-up **eTable 16.** Sensitivity Analyses Assessing the Impact of Unmeasured Confounder on the Risk of Opioid-Related Adverse Events Associated With Daily Opioid Use and Daily Opioid Dose **eTable 17.** Sensitivity Analyses Looking at the Risk of Opioid-Related Adverse Events Such as Fractures and Dizziness, Which Led to an ED Visit or Re-admission Associated With Daily Opioid Use and Daily Opioid Dose **eFigure 2.** Flowchart of Eligible Patients
