# Supplementary material for: Association of Opioid Consumption Profiles After Hospitalization With Risk of Adverse Health Care Events
Source: JAMA Netw Open. 2021 May 18;4(5):e218782. doi: 10.1001/jamanetworkopen.2021.8782 (PMC8132136; doi:10.1001/jamanetworkopen.2021.8782)
Supplement: Supplement. — eTable 1. ICD-9-CM and ICD-10-CM Codes for Opioid Abuse eTable 2. ICD-9-CM Codes for Opioid Dependence eTable 3. ICD-9-CM Codes for Adverse Effects of Opioids eTable 4. ICD-9-CM Codes for Opioid Poisoning eTable 5. ICD-9-CM Codes for Other Most Commonly Occurring Adverse Events Associated With Opioid Use eMethods 1. ATC Codes Used to Identify Opioids: N02A (Opioids), R05DA (Opium Alkaloids and Derivatives) eMethods 2. Daily Dose Calculation eTable 6. Opioid Morphine Equivalent Conversion Factor eTable 7. Description of Available Data on Drug, Patient, Provider and System Level Characteristics eFigure 1. Operational Definitions of Opioid Use Duration eMethods 3. Operational Definitions of Opioid Use Durations eTable 8. Overall Characteristics of the Opioid Prescriptions Dispensed by Patients According to Opioid Type and Potency eTable 9. Sensitivity Analyses Excluding Patients With More Than Three Opioids Dispensations in the One Year Before Initial Hospital Admission (Final Cohort N=1468) eTable 10. Sensitivity Analyses Excluding Patients With an Opioids Dispensation in the One Year Before Initial Hospital Admission (Final Cohort N = 884) eTable 11. Breakdown of the Reasons for the Healthcare Encounters in the One-Year Post-Discharge Among Patients With At Least One Opioid Dispensation eTable 12. Analyses for Current Opioid Use and Cumulative Duration of Opioid Use Based on Age eTable 13. Analyses for Current Opioid Use and Cumulative Duration of Opioid Use Based on Treatment Indication eTable 14. Results from Statistically Significant Additional Interactions Terms Between Current and Cumulative Duration of Opioid Use and Concurrent Use of Buprenorphine/Methadone and Benzodiazepines eTable 15. Characteristics of Patients in the Weighted Study Population According to the Receipt of an Opioid Dispensation at 10 Days Since Beginning of Follow-up eTable 16. Sensitivity Analyses Assessing the Impact of Unmeasured Confounder on the Risk of Opioid-Related Adverse Events As [file jamanetwopen-e218782-s001.pdf]

Supplementary Online Content

Kurteva S, Abrahamowicz M, Gomes T, Tamblyn R. Association of opioid consumption profiles after hospitalization with risk of adverse health care events. *JAMA Netw Open*. 2021;4(5):e218782. doi:10.1001/jamanetworkopen.2021.8782

eTable 1. ICD-9-CM and ICD-10-CM Codes for Opioid Abuse

eTable 2. ICD-9-CM Codes for Opioid Dependence

eTable 3. ICD-9-CM Codes for Adverse Effects of Opioids

eTable 4. ICD-9-CM Codes for Opioid Poisoning

eTable 5. ICD-9-CM Codes for Other Most Commonly Occurring Adverse Events Associated With Opioid Use

eMethods 1. ATC Codes Used to Identify Opioids: N02A (Opioids), R05DA (Opium Alkaloids and Derivatives)

eMethods 2. Daily Dose Calculation

eTable 6. Opioid Morphine Equivalent Conversion Factor

eTable 7. Description of Available Data on Drug, Patient, Provider and System Level Characteristics

eFigure 1. Operational Definitions of Opioid Use Duration

eMethods 3. Operational Definitions of Opioid Use Durations

eTable 8. Overall Characteristics of the Opioid Prescriptions Dispensed by Patients According to Opioid Type and Potency

© 2021 Kurteva S et al. *JAMA Network Open.*

eTable 9. Sensitivity Analyses Excluding Patients With More Than Three Opioids Dispensations in the One Year Before Initial Hospital Admission (Final Cohort N=1468)

eTable 10. Sensitivity Analyses Excluding Patients With an Opioids Dispensation in the One Year Before Initial Hospital Admission (Final Cohort N = 884)

eTable 11. Breakdown of the Reasons for the Healthcare Encounters in the One-Year Post-Discharge Among Patients With At Least One Opioid Dispensation

eTable 12. Analyses for Current Opioid Use and Cumulative Duration of Opioid Use Based on Age

eTable 13. Analyses for Current Opioid Use and Cumulative Duration of Opioid Use Based on Treatment Indication

eTable 14. Results from Statistically Significant Additional Interactions Terms Between Current and Cumulative Duration of Opioid Use and Concurrent Use of Buprenorphine/Methadone and Benzodiazepines

eTable 15. Characteristics of Patients in the Weighted Study Population According to the Receipt of an Opioid Dispensation at 10 Days Since Beginning of Follow-up

eTable 16. Sensitivity Analyses Assessing the Impact of Unmeasured Confounder on the Risk of Opioid-Related Adverse Events Associated With Daily Opioid Use and Daily Opioid Dose

eTable 17. Sensitivity Analyses Looking at the Risk of Opioid-Related Adverse Events Such as Fractures and Dizziness, Which Led to an ED Visit or Re-admission Associated With Daily Opioid Use and Daily Opioid Dose

eFigure 2. Flowchart of Eligible Patients

This supplementary material has been provided by the authors to give readers additional information about their work.

© 2021 Kurteva S et al. *JAMA Network Open.*

eTable 1. ICD-9-CM and ICD-10-CM Codes for Opioid Abuse

ICD-9-CM Code	Description
30550	Opioid abuse, unspecified
30551	Opioid abuse, continuous
30552	Opioid abuse, episodic

© 2021 Kurteva S et al. *JAMA Network Open.*

eTable 2. ICD-9-CM Codes for Opioid Dependence

ICD-9-CM Code	Description
30400	Opioid type dependence, unspecified
30401	Opioid type dependence, continuous
30402	Opioid type dependence, episodic
30470	Combinations of opioid type drug with any other drug dependence, unspecified
30471	Combinations of opioid type drug with any other drug dependence, continuous
30472	Combinations of opioid type drug with any other drug dependence, episodic

© 2021 Kurteva S et al. *JAMA Network Open.*

eTable 3. ICD-9-CM Codes for Adverse Effects of Opioids

ICD-9-CM Code	ICD-10-CM Code	Description
**Adverse Effects of Opioids**		
E9350		Heroin causing adverse effects in therapeutic use
E9351		Methadone causing adverse effects in therapeutic use
E9352		Other opiates and related narcotics causing adverse effects in therapeutic use
E9401		Adverse effects of opiate antagonists

© 2021 Kurteva S et al. *JAMA Network Open.*

eTable 4. ICD-9-CM Codes for Opioid Poisoning

ICD-9-CM Code	Description
96500	Poisoning by opium (alkaloids), unspecified
96501	Poisoning by heroin
96502	Poisoning by methadone
96509	Poisoning by other opiates and related narcotics
9701	Poisoning by opiate antagonists
E8500	Accidental poisoning by heroin
E8501	Accidental poisoning by methadone
E8502	Accidental poisoning by other opiates and related narcotics

© 2021 Kurteva S et al. *JAMA Network Open.*

eTable 5. ICD-9-CM Codes for Other Most Commonly Occurring Adverse Events Associated With Opioid Use

ICD-9-CM Code	Description
56400	Constipation, unspecified
54601	Slow transit constipation
54602	Outlet dysfunction constipations
56409	Other constipation
78040	Dizziness
78701	Nausea with vomiting
78702	Nausea alone
78703	Vomiting alone
80X, 81X, 82X	Fractures

eMethods 1. ATC Codes Used to Identify Opioids: N02A (Opioids), R05DA (Opium Alkaloids and Derivatives)

Exclusions: Not all drug forms were included in the analyses. Only patches and tablets of these medications were kept. Injectable, liquid and rectal forms were excluded. Methadone and buprenorphine/naloxone combinations were kept to define subclinical patient populations but were excluded from all dosing/duration calculations as these medications are used to treat addiction and we want to focus on the association of duration/dose of opioids used for pain relief.

© 2021 Kurteva S et al. *JAMA Network Open.*

eMethods 2. Daily Dose Calculation

The daily dose of each opioid was calculated by first dividing the quantity of units dispensed by the prescription duration to determine the number of units per day, and then multiplying the number of units by the strength. To account for concurrent prescriptions, a subsequent dispensation was considered as an early refill if days of overlap were ≤30% of the previous dispensation duration. Otherwise, the opioids were considered to be taken simultaneously. Daily dose of each dispensation was converted to MME doses using the Center for Disease Control Opioid Morphine Equivalent Conversion Factor and the opioid doses determined to be concurrently dispensed were added together.

© 2021 Kurteva S et al. *JAMA Network Open.*

eTable 6. Opioid Morphine Equivalent Conversion Factor [1]

Drug Name	Conversion Factor
Buprenorphine patch[2]	12.6
Buprenorphine tab or film	10
Butorphanol	7
Codeine	0.15
Dihydrocodeine	0.25
Fentanyl buccal or SL tablets, or lozenge/troche[3]	0.13
Fentanyl film or oral spray[4]	0.18
Fentanyl nasal spray[5]	0.16
Fentanyl patch[6]	7.2
Hydrocodone	1
Hydromorphone	4
Levorphanol tartrate	11
Meperidine hydrochloride	0.1
Methadone	3
Morphine	1
Nalbuphine	1
Opium	1
Oxycodone	1.5
Oxymorphone	3
Pentazocine	0.37
Tapentadol	0.4
Tramadol	0.1

[1] Centers for Disease Control and Prevention, Atlanta, GA, May 2014.
[2] The MME conversion factor for buprenorphine patches is based on the assumption that one milligram of parenteral buprenorphine is equivalent to 75 milligrams of oral morphine and that one patch delivers the dispensed micrograms per hour over a 24-hour day. Example: 5 ug/hr buprenorphine patch * 24 hrs = 120 ug/day buprenorphine = 0.12 mg/day buprenorphine = 9 mg/day oral morphine milligram equivalent. In other words, the conversion factor not accounting for days of use would be 9/5 or 1.8. However, since the buprenorphine patch remains in place for 7 days, we have multiplied the conversion factor by 7 (1.8 X 7 = 12.6). In this example, MME/day for four 5 µg/hr buprenorphine patches dispensed for use over 28 days would work out as follows: Example: 5 ug/hr buprenorphine patch * (4 patches/28 days) * 12.6 = 9 MME/day.
[3] The MME conversion factor for fentanyl buccal tablets, sublingual tablets, and lozenges/troche is 0.13. This conversion factor should be multiplied by the number of micrograms in a given lozenge/troche.
[4] The MME conversion factor for fentanyl film and oral spray is 0.18. This reflects a 40% greater bioavailability for films compared to lozenges/tablets and 38% greater bioavailability for oral sprays compared to lozenges/tablets.
[5] The MME conversion factor for fentanyl nasal spray is 0.16, which reflects a 20% greater bioavailability for sprays compared to lozenges/tablets.
[6] The MME conversion factor for fentanyl patches is based on the assumption that one milligram of parenteral fentanyl is equivalent to 100 milligrams of oral morphine and that one patch delivers the dispensed micrograms per hour over a 24 hour day. Example: 25 ug/hr fentanyl patch * 24 hrs = 600 ug/day fentanyl = 60 mg/day oral morphine milligram

© 2021 Kurteva S et al. *JAMA Network Open.*

equivalent. In other words, the conversion factor not accounting for days of use would be 60/25 or 2.4. However, since the fentanyl patch remains in place for 3 days, we have multiplied the conversion factor by 3 (2.4 X 3 = 7.2).
In this example, MME/day for ten 25 µg/hr fentanyl patches dispensed for use over 30 days would work out as follows: Example: 25 ug/hr fentanyl patch * (10 patches/30 days)* 7.2 = 60 MME/day.

Sources:

1) Centers for Medicare & Medicaid Services. Opioid Oral Morphine Milligram Equivalent (MME) Conversion Factors. https:\\www.cms.govMedicarePrescription-Drug-CoveragePrescriptionDrugCovContraDownloadsOpioid-Morphine-EQConversion-Factors-vFeb-.pdf. Accessed: September 5, 2019
2) Svendsen, K., Borchgrevink, P., Fredheim, O., Hamunen, K., Mellbye, A., & Dale, O. (2011). Choosing the unit of measurement counts: the use of oral morphine equivalents in studies of opioid consumption is a useful addition to defined daily doses. Palliative Medicine, 25(7), 725–732. http://doi.org/10.1177/0269216311398300

© 2021 Kurteva S et al. *JAMA Network Open.*

eTable 7. Description of Available Data on Drug, Patient, Provider and System Level Characteristics

	Description	Measurement	Timing of Measurement	Functional Form
**Opioid-related Characteristics**				
Opioid Dispensations				
ATC code	Anatomical Therapeutic Chemical Classification System code used to identify opioids and other concurrent medications that the patient is taking<br>**Opioids ATC Included: N02A, R05DA**	RAMQ prescription claims.	In the community one year prior to admission and one year post-discharge	N/A
Dose	The daily amount of drug taken by patient will be calculated based on information about the number of tablets prescribed, strength and number of days' supply; daily dose will be converted to milligram morphine equivalents to facilitate comparisons across opioids.	From RAMQ prescription claims	In the community one year prior to admission and one year post-discharge	Continuous, categorical, time-varying
Duration	The days' supply on the drug claim as entered by the pharmacist	From RAMQ prescription claims	In the community one year prior to admission and one year post-discharge	Continuous, categorical, time-varying
Type of opioid	Type of opioid ingredient. E.g; Hydromorphone, oxycodone, morphine, fentanyl, etc.	From RAMQ prescription claims	One year post-discharge	Categorical, time-varying
Opioid Administration in Hospital				
ATC code	Anatomical Therapeutic Chemical Classification System code used to identify administered opioids	Hospital pharmacy	In hospital	Categorical

© 2021 Kurteva S et al. *JAMA Network Open.*

Opioid Prescription at Hospital Discharge				
Status of opioid medication	Continued or stopped from community, newly prescribed at discharge	From patient chart	At hospital discharge	Categorical, time-fixed
Reason for opioid prescribing	Pain-related including having had surgery as well as other diagnoses such as having insomnia or anxiety as recorder during the hospitalization	From patient chart	In-hospital	Categorical, time-fixed
Presence of a multi-modal pain management regimen	The opioid prescription at hospital discharge was part of multi-modal pain treatment regimen	From patient chart	At hospital discharge	Categorical, time-fixed
Patient-reported adherence to opioid prescription given at hospital discharge	Whether patient takes the medication as prescribed or deviated from the prescription posology (e.g.; medication taken less or more often than directed to patient due to pain complaints, complications, side-effects, etc.)	RAMQ prescription claims to determine whether opioid was filled post-discharge; Patient interview to assess if patients are taking the drugs as prescribed	30-days post-hospital discharge	Categorical, time-fixed
**Patient-level Characteristics**				
Demographics				
Age		From patient chart	Admission to hospital	Continuous, time-varying
Sex	Male, Female	From patient chart	Admission to hospital	Binary, time-fixed
Drug insurance status	E.g.; Full copay, partial copay, no copay Serves as proxy for socio-economic status.	From RAMQ drug programs	Admission to hospital	Categorical, time-fixed
Co-Existing Illnesses				
History of mental health conditions	E.g.; Anxiety, depression, psychiatric diagnosis, mood disorder, and post-traumatic stress disorder	ICD-9 from RAMQ medical services and ICD-10 codes from hospitalization	In community one year prior to admission, in hospital, post-discharge	Binary per condition, time-varying

© 2021 Kurteva S et al. *JAMA Network Open.*

		data		
Pain syndromes	E.g.; Chronic back pain, back and neck pain, back disorder, arthritis, migraine, headache, fibromyalgia, fracture	ICD-9 from RAMQ medical services and ICD-10 codes from hospitalization data	In community one year prior to admission, in hospital, post-discharge	Binary per condition, time-varying
Health conditions Associated with abuse	E.g.; Alcohol abuse, drug abuse	From patient chart. Also from RAMQ medical series and prescription claims	In community one year prior to admission, in hospital, post-discharge	Binary per condition, time-varying
Tobacco use	Patient-reported history of tobacco use	From hospital charts	At admission	Binary, time-fixed
Cancer diagnosis	E.g.; Metastatic, Non-metastatic, Lymphoma	ICD-9 from RAMQ medical services and ICD-10 codes from hospitalization data	In community one year prior to admission, in hospital, post-discharge	Binary per condition, time-varying
Other comorbidities	E.g.; Acute MI, cerebrovascular diseases, chronic kidney, COPD, diabetes, heart failure, hypertension, ischemic heart disease, liver, obesity	ICD-9 from RAMQ medical services and ICD-10 codes from hospitalization data	In community one year prior to admission, in hospital, post-discharge	Binary per condition, time-varying
Drug and Healthcare Utilization				
Use of potential interacting drugs increasing the risk of opioid misuse	E.g.; Selective serotonin reuptake inhibitors, other antidepressants, benzodiazepines, other antipsychotic drugs, central nervous system depressants, psychotropic medication	ATC codes, DIN, Generic Drug name used to extract information from RAMQ prescription claims, hospital data, patient chart.	In community one year prior to admission, in-hospital, post-discharge	Binary per drug, time-varying
Use of non-opioid pain	E.g.; NSAIDS, COX-2, Acetaminophen, Gabapentin,	ATC codes, DIN, Generic	In community one year prior to	Binary per drug, time-

© 2021 Kurteva S et al. *JAMA Network Open.*

medications	anti-migraine medications, muscle-relaxants, other anti-inflammatories and anti-rheumatoid medications	Drug name used to extract information from RAMQ prescription claims, hospital data, patient chart.	admission, in-hospital, post-discharge	varying
Number of ED visits and hospitalizations	Total number of ED visits and hospitalizations	From RAMQ prescription claims and hospital data	One year prior to hospital admission & one year post-discharge	Categorical, continuous, time-varying
Measures of Care Continuity				
Number of physicians	Number of unique physicians that prescribed an opioid medication to a patient in the year post hospital admission	From RAMQ medical services	One year prior to hospital admission & one year post-discharge	Categorical, continuous, cumulative time-varying
Number of dispensing pharmacies	Number of unique pharmacies that a patient has opioid medications dispensed at in the one year post to hospital admission	From RAMQ medical services	One year prior to hospital admission & One year post-discharge	Categorical, continuous, cumulative time-varying
Other Patient Drug Behavior Characteristics				
Time since hospital discharge	The time elapsed between patent's hospital discharge and their first opioid dispensation	From RAMQ prescription claims and hospital data	One year post-discharge	Continuous, time-fixed
Discontinuation of opioid use	Recent discontinuation of opioid use in the past 2 weeks	From RAMQ medical services	One year post-discharge	Categorical, time-varying
Daily opioid dose increase	Recent increase in the daily opioid use in the past 2 weeks	From RAMQ medical services	One year post-discharge	Categorical, time-varying
Add-on opioid	Recent add-on of another opioid type in the past 2 weeks	From RAMQ medical services	One year post-discharge	Categorical, time-varying
**In-hospital Characteristics**				
Hospital patient is admitted to	Montreal General or Royal Victoria hospital	From hospital chart	Upon admission to the hospital	Binary, time-fixed
Hospital unit the patient is admitted to	Medical or surgical unit	From hospital chart	Upon admission to the hospital	Binary, time-fixed

© 2021 Kurteva S et al. *JAMA Network Open.*

Reason for index hospital admission	Reasons were classified as opioid-related if patient presented to the hospital for an opioid-related disorder, poisoning by opioids, or fractures.	From hospitalization data	During the hospital stay	Binary, time-fixed
Discharge Destination	Home community, long term care	Patient chart	Upon discharge	Binary, time-fixed
RightRx patients	Patients, who were part of the initial randomized controlled trial	Patient chart	Upon discharge	Binary, time-fixed

© 2021 Kurteva S et al. *JAMA Network Open.*

eFigure 1. Operational Definitions of Opioid Use Duration

1.a. Definition of opioid exposure based on cumulative duration of opioid use.

© 2021 Kurteva S et al. *JAMA Network Open.*

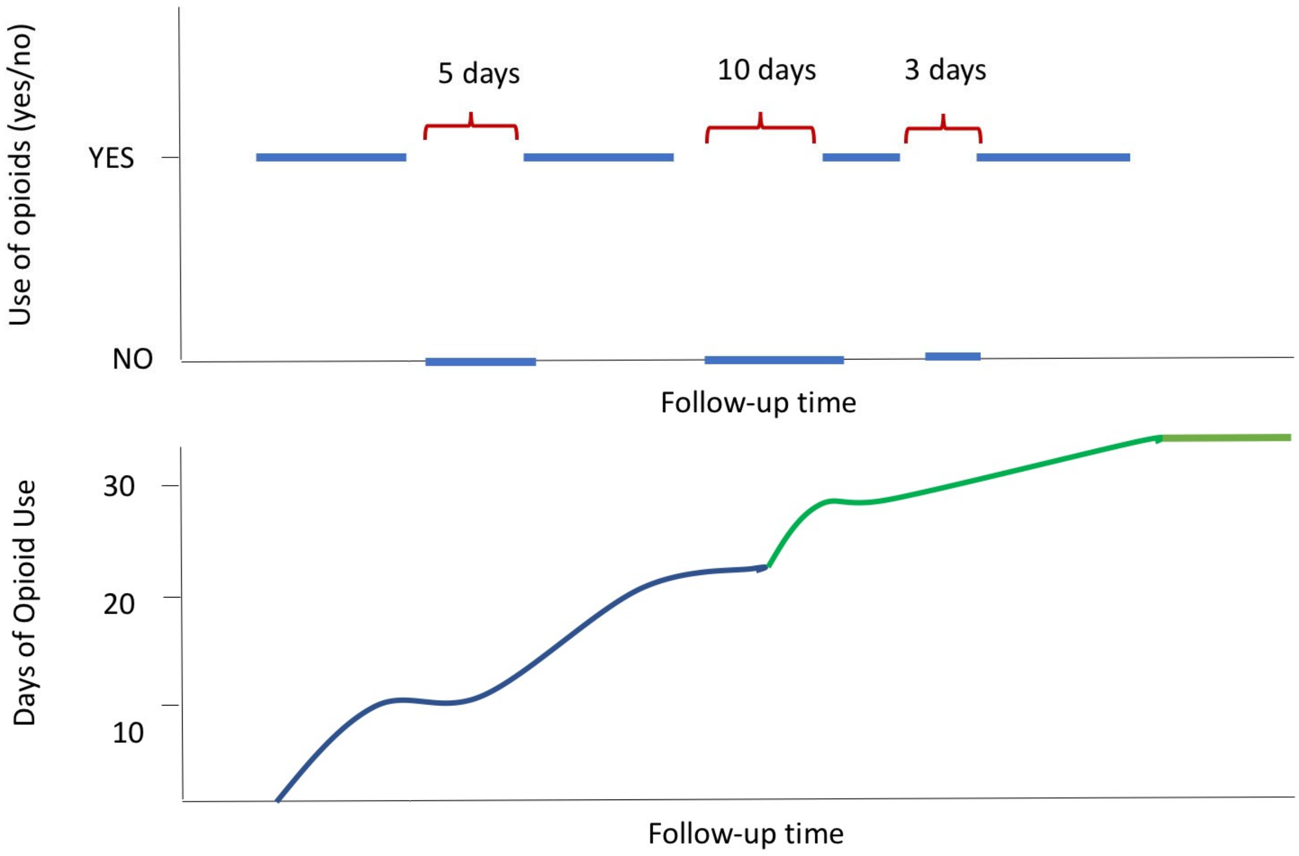

© 2021 Kurteva S et al. *JAMA Network Open.*

1.b. Definition of opioid exposure based on continuous duration of opioid use.

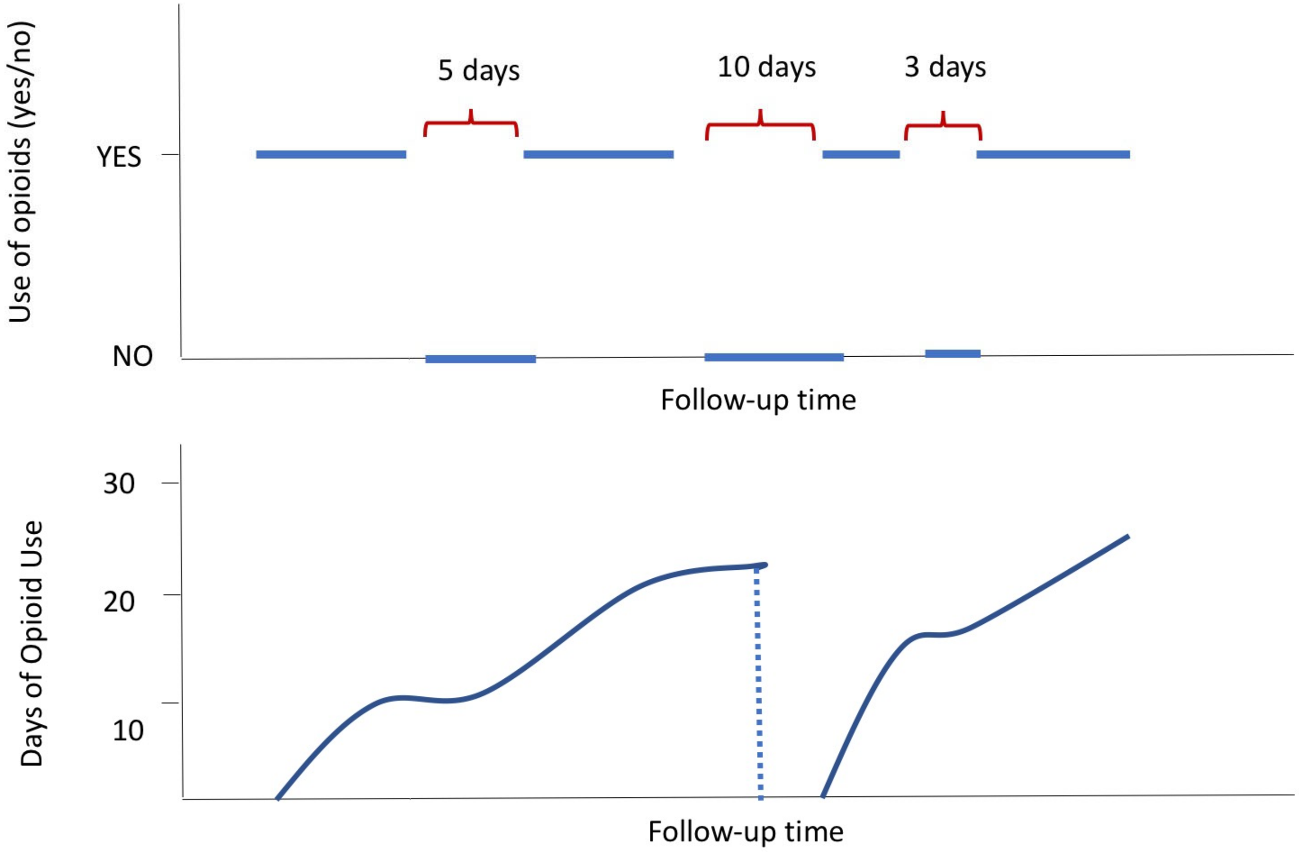

© 2021 Kurteva S et al. *JAMA Network Open.*

eMethods 3. Operational Definitions of Opioid Use Durations

Cumulative duration of past use assessed the long-term impact of opioids, where the effect on the outcome persisted upon discontinuation: defined as the total number of days exposed, calculated by summing the durations of all dispensations between cohort entry (first opioid dispensation) and a given day during the follow-up. Cumulative users represented patients who used opioids only when needed, thus accumulating use over time. On the other hand, we assessed continuous duration where the effect of opioids accumulated by dispensation supply but the risk returned to baseline after discontinuation. Continuous duration was defined similarly but was allowed to increase only during the periods of un-interrupted use and was reset to zero if there was a gap of >5 days between subsequent dispensations.

© 2021 Kurteva S et al. *JAMA Network Open.*

eTable 8. Overall Characteristics of the Opioid Prescriptions Dispensed by Patients According to Opioid Type and Potency

**Ingredient** *(molecule)*	**Person-Months ***	**Quantity** *Mean (SD), Median, IQR*	**Days' Supply** *Mean (SD), Median, IQR*	**Daily Dose** *Mean (SD), Median, IQR*
Codeine	29.6	52.8 (44.9), 30.0, 16.0 – 70.0	14.9 (11.8), 8.0, 6.0 – 31.0	20.9 (11.0), 17.7, 14.5 – 26.3
Morphine	31.5	31.9 (31.7), 21.0, 7.0 – 50.0	11.4 (9.9), 8.0, 6.0 – 11.0	29.9 (31.4), 18.8, 15.0 – 35.0
Oxycodone	32.9	41.0 (41.6), 30.0, 14.0 – 60.0	12.1 (9.8), 8.0, 6.0 – 15.0	40.4 (35.7), 30.0, 21.8 – 52.5
Hydromorphone	28.9	37.5 (42.7), 21.0, 12.5 -60	10.3 (9.3), 8.0, 5.0 – 11.0	59.2 (65.2), 32.0, 20.6 – 69.7
Fentanyl	32.1	8.5 (4.5), 10.0, 5.0-10.0	22.0 (9.4), 27.0, 16.0 – 31.0	108.4 (110.9), 58.1, 27.9 – 168.7

*per 1000 per month; person-month represents person-time from discharge until last date when patients had a supply for that drug during the follow-up period.

© 2021 Kurteva S et al. *JAMA Network Open.*

eTable 9. Sensitivity Analyses Excluding Patients With More Than Three Opioids Dispensations in the One Year Before Initial Hospital Admission (Final Cohort N=1468)

**Opioid Exposure Metric**	**HR Stabilized Weights**	**95% CI**
**Current Opioid Use**		
No use	Ref	Ref
Use	1.76	1.23 – 2.52
**Cumulative Duration of Opioid Use**		
1-30	Ref	Ref
30-60	1.53	0.98 – 2.40
60-90	2.15	1.15 – 4.02
>90	2.60	1.57 – 4.30

© 2021 Kurteva S et al. *JAMA Network Open.*

eTable 10. Sensitivity Analyses Excluding Patients With an Opioid Dispensation in the One Year Before Initial Hospital Admission (Final Cohort N = 884)

**Opioid Exposure Metric**	**HR Stabilized Weights**	**95% CI**
**Current Opioid Use**		
Non-users	Ref	Ref
Users	2.40	1.31 – 4.41
**Cumulative Duration of Opioid Use**		
1-30	Ref	Ref
30-60	1.05	0.49 – 2.22
60-90	1.43	0.46 – 4.44
>90	9.46	4.69 – 19.08

© 2021 Kurteva S et al. *JAMA Network Open.*

eTable 11. Breakdown of the Reasons for the Healthcare Encounters in the One-Year Post-Discharge Among Patients With At Least One Opioid Dispensation

	**N (%) ***
Drug dependence, morphine type	2 (3.9)
Fractures	219 (51.8)
General symptoms, dizziness and giddiness	78 (18.4)
Functional digestive disorders, constipation	58 (13.7)
Symptoms involving digestive system, nausea and vomiting	66 (15.6)

* Total number of reasons does not equal the total number of patients as multiple diagnoses may have been recorded per patient at admission.

© 2021 Kurteva S et al. *JAMA Network Open.*

eTable 12. Analyses for Current Opioid Use and Cumulative Duration of Opioid Use Based on Age

Opioid Exposure Metric	Average Starting Dose (MME) Mean (SD)	Average Daily Dose (MME) Mean (SD)	HR Stabilized Weights	95% CI
**Current Opioid Use**				
Patients ≤64 years of age				
No use	34.9 (21.9)	-	Ref	-
Use	49.9 (51.9)	71.8 (91.1)	3.38	1.60 – 7.13
Patients >64 years of age				
No use	32.8 (17.9)	-	Ref	-
Use	35.9 (36.0)	48.1 (61.7)	2.01	1.20 – 2.34
**Cumulative Duration of Opioid Use**				
Patients ≤64 years of age				
1-30	41.2 (36.3)	49.2 (55.8)	Ref	-
30-60	47.4 (46.8)	67.3 (88.3)	2.79	1.24 – 6.28
60-90	49.9 (49.1)	76.6 (97.8)	0.65	0.13 – 3.24
>90	57.7 (62.4)	89.8 (107.3)	3.23	1.15 – 9.44
Patients >64 years of age				
1-30	33.5 (28.3)	37.4 (35.9)	Ref	-
30-60	35.1 (34.9)	42.1 (58.1)	1.54	0.84 – 2.82
60-90	27.2 (40.8)	54.1 (74.5)	4.31	2.08 – 8.93
>90	38.6 (42.1)	60.3 (77.1)	5.29	2.29 – 12.2

Note: Results in the Appendix were presented for all interactions, regardless of statistical significance, as, in previous research, these subgroups have been studied separately and having distinct estimates could serve to provide meaningful comparisons across these subclinical populations. The two-way interaction terms p-values with current opioid use and age (0.22); the two-way overall interaction terms p-values with cumulative duration of opioid use and age were 0.05. With respect to the various categories of cumulative duration of use, the respective p-values were the following: 0.24 (30-60 days), 0.03 (60-90 days), 0.41 (>90 days).

© 2021 Kurteva S et al. *JAMA Network Open.*

eTable 13. Analyses for Current Opioid Use and Cumulative Duration of Opioid Use Based on Treatment Indication

Opioid Exposure Metric	Average Starting Dose (MME) Mean (SD)	Average Daily Dose (MME) Mean (SD)	HR Stabilized Weights	95% CI
**Current Opioid Use**				
Medical Patients				
No use	25.9 (27.0)	-	Ref	-
Use	45.1 (58.4)	46.6 (53.9)	1.10	0.65 – 1.88
Surgical Patients				
No use	37.8 (17.3)	-	Ref	-
Use	37.6 (20.5)	68.0 (91.0)	3.55	1.82 – 6.85
Non-cancer patients				
No use	29.5 (18.3)	-	Ref	
Use	38.9 (48.9)	53.9 (77.2)	1.89	1.02 – 3.49
Cancer patients				
No use	38.8 (48.8)	-	Ref	
Use	43.6 (36.5)	59.9 (72.4)	1.58	0.87 – 2.87
**Cumulative Duration of Opioid Use**				
Medical Patients				
1-30	36.7 (49.7)	47.1 (63.9)	Ref	-
30-60	41.6 (53.8)	60.1 (83.2)	1.87	0.98 – 3.53
60-90	44.2 (56.9)	69.5 (92.6)	1.73	0.68 – 4.42
>90	50.2 (62.9)	79.7 (101.1)	2.26	0.94 – 5.43
Surgical Patients				
1-30	35.9 (18.8)	38.9 (31.5)	Ref	-
30-60	38.3 (20.9)	44.0 (59.3)	1.22	0.55 – 2.69
60-90	39.9 (20.9)	54.8 (74.1)	4.81	1.76 – 13.1

© 2021 Kurteva S et al. *JAMA Network Open.*

>90	39.9 (22.7)	59.7 (71.2)	7.80	3.20 – 19.1
Non-cancer Patients				
1-30	33.0 (35.8)	38.5 (47.6)	Ref	-
30-60	38.1 (47.4)	55.0 (84.7)	1.53	0.72 – 3.27
60-90	40.4 (51.8)	61.6 (90.6)	2.25	0.76 – 6.64
>90	44.4 (58.0)	66.9 (90.4)	2.25	0.78 – 6.50
Cancer Patients				
1-30	39.3 (25.8)	44.5 (39.7)	Ref	-
30-60	41.6 (32.5)	48.9 (58.4)	1.85	0.98 – 3.49
60-90	44.1 (36.2)	64.2 (79.4)	3.26	1.36 – 7.81
>90	48.6 (45.5)	78.3 (93.1)	4.43	1.85 – 10.6

Note: Results in the Appendix were presented for all interactions, regardless of statistical significance, as, in previous research, these subgroups have been studied separately and having distinct estimates could serve to provide meaningful comparisons across these subclinical populations. The two-way interaction terms p-values with current opioid use and discharged unit (0.003), cancer diagnoses (0.68); the two-way overall interaction terms p-values with cumulative duration of opioid use and discharged unit were 0.03 and with cancer diagnoses**,** 0.65. Despite the overall-value for the interaction between hospital discharge unit and cumulative duration of use, only the duration of more than 90 days showed to be significant (p-value = 0.02) as such, only results for this subclinical category were presented, along with the estimate for current opioid use, which also tested significant.

© 2021 Kurteva S et al. *JAMA Network Open.*

eTable 14. Results from Statistically Significant Additional Interactions Terms Between Current and Cumulative Duration of Opioid Use and Concurrent Use of Buprenorphine/Methadone and Benzodiazepines

Opioid Exposure Metric	Average Starting Dose (MME) Mean (SD)	Average Daily Dose (MME) Mean (SD)	HR Stabilized Weights	95% CI
**Current Opioid Use**				
No concurrent use with buprenorphine/methadone				
No use	33.4 (19.1)	-	Ref	-
Use	41.0 (43.3)	56.6 (74.9)	1.76	1.07 – 2.89
Concurrent use with buprenorphine/methadone				
No current daily opioid use	85.8 (51.7)	-	Ref	-
Current daily opioid use	52.1 (34.9)	81.1 (73.9)	0.08	0.01 – 1.21
**Cumulative Duration of Opioid Use**				
No concurrent use with benzodiazepines				
1-30	36.1 (31.2)	41.4 (43.9)	Ref	-
30-60	39.9 (40.1)	51.8 (72.0)	1.57	0.92 – 2.68
Concurrent use with benzodiazepines				
1-30	38.1 (41.6)	42.7 (48.6)	Ref	-
30-60	38.3 (46.9)	53.3 (79.8)	6.81	1.89 – 24.6

Note: The results in main text as well as in the Appendix table were presented only for interaction terms which were significant. For example, the likelihood ratio test for the interaction between current daily opioid use and opioid formulation had a p-value >0.05 and thus, results were not shown. The corresponding p-values for the two-way interaction terms between current opioid use and use of methadone/buprenorphine (0.03); the p-value for the two-way interaction terms between cumulative duration of opioid use of benzodiazepines (0.045). Despite the overall significant p-value for the interaction between concurrent users of benzodiazepines and cumulative duration of use, only the duration between 30-60 days of use showed to be significant for concurrent opioid and benzodiazepine use (p-value = 0.035) as such, only results for this subclinical category were presented.

© 2021 Kurteva S et al. *JAMA Network Open.*

eTable 15. Characteristics of Patients in the Weighted Study Population According to the Receipt of an Opioid Dispensation at 10 Days Since Beginning of Follow-up

**Characteristic**	**No Opioid Dispensation**	**Opioid Dispensation**	**Absolute Standardized Difference**
Age, mean	67.1(13.6)	67.1 (13.2)	0.001
Male	60.40	58.92	0.03
Length of hospital stay (≥ 6 days)	77.15	82.27	0.13
**Hospital unit**			
Internal medicine	24.48	28.46	0.09
Cardiac Surgery	51.05	30.77	0.41
Thoracic surgery	24.47	40.77	0.35
**Healthcare Utilization**			
Number of dispensing pharmacies (>1)	0.3465	0.3257	0.04
Radiotherapy	0.1044	0.1500	0.13
Chemotherapy	0.1220	0.1836	0.17
**Pain Regiment at Discharge**			
Opioids	0.7980	0.7810	0.04
Analgesics	0.8433	0.7950	0.12
In-Hospital Medication Use			
Antidepressants	0.1138	0.1785	0.18
Opioids	0.9247	0.9333	0.03
Benzodiazepines	0.8131	0.7704	0.10
Analgesics	0.9624	0.9691	0.03
**Medication Use**			
History of opioid use	0.2912	0.4426	0.31

© 2021 Kurteva S et al. *JAMA Network Open.*

Benzodiazepines	0.0261	0.0201	0.04
Analgesics	0.0853	0.0801	0.02
Antidepressants	0.0242	0.0278	0.02
**Targeted Comorbidities**			
Cancer	0.6672	0.6191	0.10
Mental illness	0.1730	0.1553	0.05
Opioid and non-opioid substance abuse	0.0401	0.0292	0.06
Alcohol abuse	0.0179	0.0201	0.02
Pain Syndromes	0.4870	0.3975	0.18
Cardiovascular Diseases	0.5727	0.5441	0.06
Cerebrovascular Diseases	0.0905	0.1066	0.05
Chronic obstructive pulmonary disease	0.2160	0.2194	0.008

© 2021 Kurteva S et al. *JAMA Network Open.*

eTable 16. Sensitivity Analyses Assessing the Impact of Unmeasured Confounder on the Risk of Opioid-Related Adverse Events Associated With Daily Opioid Use and Daily Opioid Dose

	**ED visits/re-admission/death**	**ED visits/re-admissions**	**Death**
**Current Daily Use**			
No	Ref	Ref	Ref
Yes	1.50 (0.91 – 2.49)	1.71 (0.86 – 3.42)	1.37 (0.69 – 2.71)
**MME Daily Dose**			
≤90	Ref	Ref	Ref
>90	3.49 (1.57 – 7. 73)	1.06 (0.30 – 3.76)	5.81 (2.11 – 16.03)

© 2021 Kurteva S et al. *JAMA Network Open.*

eTable 17. Sensitivity Analyses Looking at the Risk of Opioid-Related Adverse Events Such as Fractures and Dizziness, Which Led to an ED Visit or Re-admission Associated With Daily Opioid Use and Daily Opioid Dose

**Opioid Exposure Metric**	**Fracture-related events**	**Fracture-related ED visits/re-admissions**	**Other opioid-related events**	**Other opioid-related resED visits/re-admissions**
**Current Opioid Use**				
No use	59	Ref	33	Ref
Use	29	1.46 (0.66 – 3.24)	26	1.69 (0.65 – 4.33)
**Cumulative Duration of Opioid Use**				
1-30	52	Ref	37	Ref
30-60	13	1.52 (0.69 – 3.33)	12	1.54 (0.60 – 3.94)
60-90	6	2.10 (0.62 – 7.10)	2	-
>90	17	2.21 (0.83 – 5.88)	8	1.60 (0.43 – 6.00)
**Continuous Duration of Opioid Use**				
0	59	Ref	33	Ref
1-30	15	1.18 (0.24 - 5.69)	20	2.58 (0.79 – 8.45)
30-60	5	3.86 (0.96 – 15.5)	2	1.76 (0.38 – 8.06)
>60	9	0.71 (0.23 – 2.16)	4	0.62 (0.13 – 3.06)
**MME Daily Dose**				
≤90	81	Ref	33	Ref
>90	7	1.88 (0.58 – 6.01)	26	0.82 (0.11 – 5.97)

© 2021 Kurteva S et al. *JAMA Network Open.*

eFigure 2. Flowchart of Eligible Patients

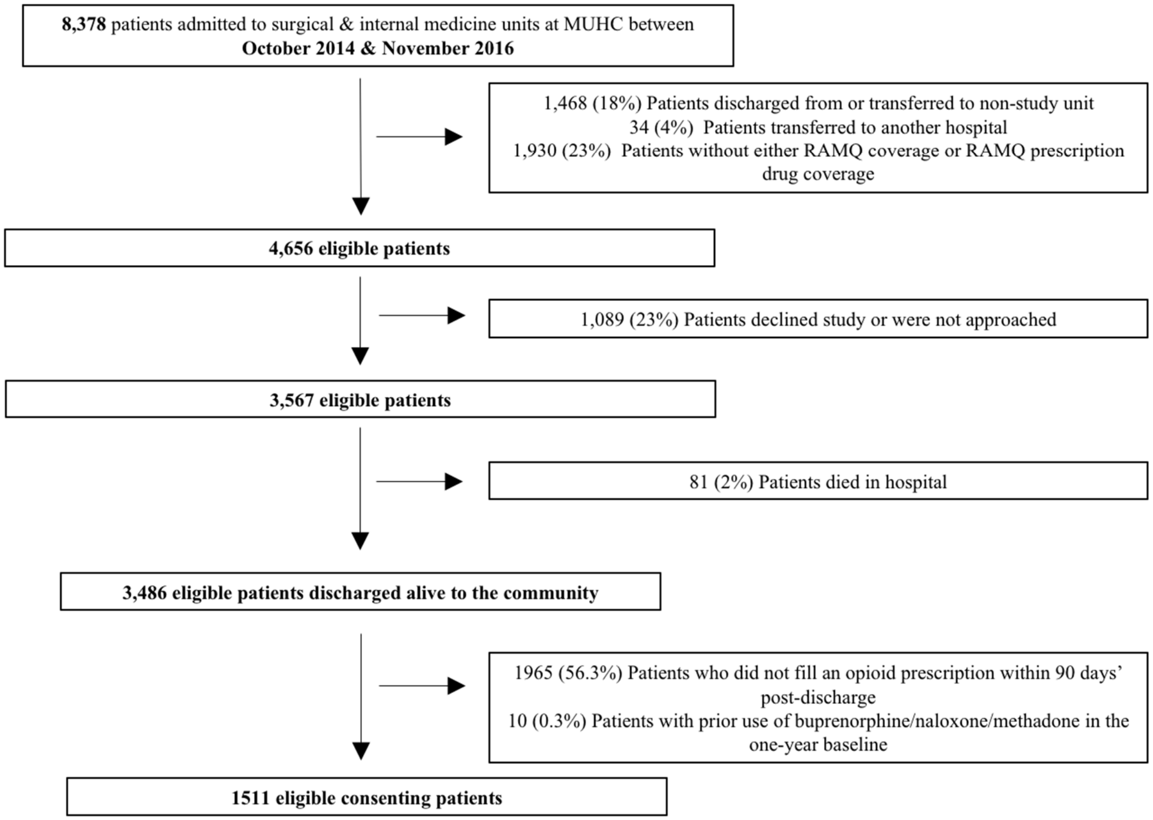

© 2021 Kurteva S et al. *JAMA Network Open.*
